# Secondary Data Analysis of Epidemiology in Asia

**DOI:** 10.2188/jea.JE20140137

**Published:** 2014-09-05

**Authors:** Yoshitaka Murakami

**Affiliations:** Professor, Department of Medical Statistics, Toho University, Tokyo, Japan

**Keywords:** secondary data, Asia, large database

It is widely acknowledged that there is great potential for utilizing databases in health care systems in developed countries.^1^ Modern health care databases have two characteristics that make them attractive to epidemiologists who work on ‘real-world’ problems: routinely conducted data collection and perpetual electronic data storage. Pharmaco-epidemiology and health services research have long taken a leading role in utilizing these kinds of databases in epidemiology, and the emerging research area called comparative effectiveness research (CER) has recently begun to utilize health care databases to weigh the benefits and harms of different interventions.

Health care databases are typically used by researchers as secondary data sources, or existing data generated for a purpose different from the research activity for which it is being used.^2^ There are pros and cons involved in the use of secondary data. From a beneficial aspect, generation of new data is often expensive and time-consuming; further, collection of new data may raise concerns about privacy and unwanted disclosure of data.^2^ Using secondary data for epidemiologic research can therefore save time and resources if the data meet the study purpose. However, while there are great advantages to the use of secondary data, bias in the database, which can be introduced through confounding, missing data, and misclassification, is a great threat to the validity of epidemiologic research.

To address these issues, propensity score matching, sensitivity analysis, and instrumental variable analysis are used to assess and control for bias in observational CER.^3^ Before applying these novel epidemiologic methods, it is crucial to check the potential for and magnitude of bias by using real-world data. As each database has its own specific purpose (e.g., patient management or health insurance claims), researchers using the databases for secondary purposes sometimes do not know the general characteristics of the databases. The descriptive information provided by databases is therefore important for conducting, analyzing, and interpreting secondary data.

Tanihara analyzed the database of health insurance claims (HIC) of the National Health Insurance Organization of Kumamoto Prefecture in May 2010.^4^ This large database included a total of 3.8 million diagnoses, comprising all computerized health insurance claims in the prefecture for inpatient or outpatient medical care and DPC/PDPS (diagnosis procedure-combination per-diem payment system). Diagnoses in the database were coded in accordance with the International Statistical Classification of Diseases and Related Health Problems, 10th Revision, but some diagnoses were left uncoded. These uncoded diagnoses are often excluded from analyses, which might introduce bias.

Tanihara stated that “the main problem of uncoded diagnoses is that the investigation of large administrative databases using HICs in Japan may have been biased if there was a tendency for one or more specific diagnoses to be uncoded more often than others”. He tackled this issue, finding that the overall proportion of uncoded diagnoses was 9.6% and that proportions differed by HIC type and disease category. Among HIC types, DPC/PDPS included more uncoded diagnoses (14.6%) than outpatient (9.3%) and inpatient (10.9%) records. Among disease categories, congenital malformation, deformations, and chromosomal abnormalities (outpatients; 19.5%); injury, poisoning, and certain other consequences of external causes (inpatients; 19.7%); and neoplasms (DPC/PDPS; 77.9%) showed high percentages of uncoded diagnoses. These findings indicate that Japanese health statistics based on computerized health insurance claims might be biased if we exclude uncoded diagnoses from the analysis.

Rapid economic growth in the Asia-Pacific region has changed population lifestyle and the environment in which we live over the past decade. Non-communicable diseases (NCDs), such as cancer, cardiovascular disease, and diabetes, are now emerging threats to public health in this region, and effective prevention measures for NCDs are needed. The analysis of ‘real-world’ data is recognized as the best means for development of an evidence-based health policy. Researchers and public health officers in Japan have drawn attention to the national integrated database of medical receipts and health checkup information, called the ‘National Database’. However, access to this database is still restricted under the current government. Miyagawa et al. analyzed part of this database (212 000 residents) and estimated age- and sex-specific prevalence, rates of treatment, and control of cardiovascular risk factors among adults in Shiga prefecture.^5^ Age- and sex-specific prevalence is a fundamental epidemiologic measure that describes health status by region, and stable estimates can only be obtained from large databases. We hope that descriptive epidemiology involving large databases will become more common in the future. We also encourage researchers to utilize large health care databases available in Asian countries.
